# Glucose metabolism and tumour microenvironment in pancreatic cancer: A key link in cancer progression

**DOI:** 10.3389/fimmu.2022.1038650

**Published:** 2022-12-12

**Authors:** Shi Dong, Wancheng Li, Xin Li, Zhengfeng Wang, Zhou Chen, Huaqing Shi, Ru He, Chen Chen, Wence Zhou

**Affiliations:** ^1^ The Second School of Clinical Medicine, Lanzhou University, Lanzhou, China; ^2^ The First School of Clinical Medicine, Lanzhou University, Lanzhou, China; ^3^ Department of General Surgery, The First Hospital of Lanzhou University, Lanzhou, China; ^4^ Department of General Surgery, Lanzhou University Second Hospital, Lanzhou, China

**Keywords:** pancreatic cancer, tumour microenvironment, glucose metabolism, treatment, prognosis

## Abstract

Early and accurate diagnosis and treatment of pancreatic cancer (PC) remain challenging endeavors globally. Late diagnosis lag, high invasiveness, chemical resistance, and poor prognosis are unresolved issues of PC. The concept of metabolic reprogramming is a hallmark of cancer cells. Increasing evidence shows that PC cells alter metabolic processes such as glucose, amino acids, and lipids metabolism and require continuous nutrition for survival, proliferation, and invasion. Glucose metabolism, in particular, regulates the tumour microenvironment (TME). Furthermore, the link between glucose metabolism and TME also plays an important role in the targeted therapy, chemoresistance, radiotherapy ineffectiveness, and immunosuppression of PC. Altered metabolism with the TME has emerged as a key mechanism regulating PC progression. This review shed light on the relationship between TME, glucose metabolism, and various aspects of PC. The findings of this study provide a new direction in the development of PC therapy targeting the metabolism of cancer cells.

## 1 Introduction

As an extremely aggressive malignancy, pancreatic cancer (PC) is becoming the second leading cause of cancer-related deaths globally (1). The lack of early diagnosis strategies limits the effective treatment of PC. Accordingly, PC has a poor prognosis, with an overall 5-year survival rate of less than 5% (2). Less than 20% of PC patients are legible for surgery, the only treatment for this cancer (3). Even so, the recurrence rate of PC after surgery remains high (4). Even though gemcitabine has been developed for treating PC, drug resistance limits its clinical application. Additionally, it does not significantly improve the overall survival of PC patients (5). Furthermore, the use of postoperative radiotherapy has not been supported by sufficient evidence. Hammel et al. found no significant difference in the median survival time of the chemoradiotherapy group (109 cases, median survival time of 15.2 months) compared with the chemotherapy group (112 cases, median survival time of 16.5 months) (P>0.05) (6). Even though targeted therapy and immunotherapy could cure PC, research on these treatment options is still at infancy (7). Overall, the path to curing PC remains arduous.

The aggressive proliferation of tumour cells is inextricably linked to the supply of energy. Metabolic reprogramming provides continuous energy for the growth and metastasis of tumour cells. Among glucose, amino acids, and lipids, glucose metabolism is the most common, which generates abundant energy, macromolecular precursors and reducing equivalents (8). Endothelial cell dysfunction induced by high metabolic rate and high oxygen consumption of tumour cells or other effects on blood vessels is a typical microenvironmental hallmark of most solid tumours, especially PC (9). However, Warburg found that under hypoxia, cancer cells still utilize glycolysis to obtain ATP, through aerobic glycolysis (Warburg effect), which is accompanied by a downregulation of the Pasteur effect. This metabolic change in the TME a combination of cancer cells and their microenvironment (10). Moreover, this metabolism increases tumour plasticity and heterogeneity, resulting in a more aggressive and metastatic phenotype (11). Aerobic glycolysis nourishes PC cells and regulates metastasis, immune evasion, chemoresistance, and radiotherapy ineffectiveness (12). An in-depth understanding of the mechanism of glucose metabolism reprogramming (glycolysis) in PC cells and the microenvironment could reveal new approaches for PC treatment.

## 2 Reprogramming of glucose metabolism in pancreatic cancer

**Figure 1:**
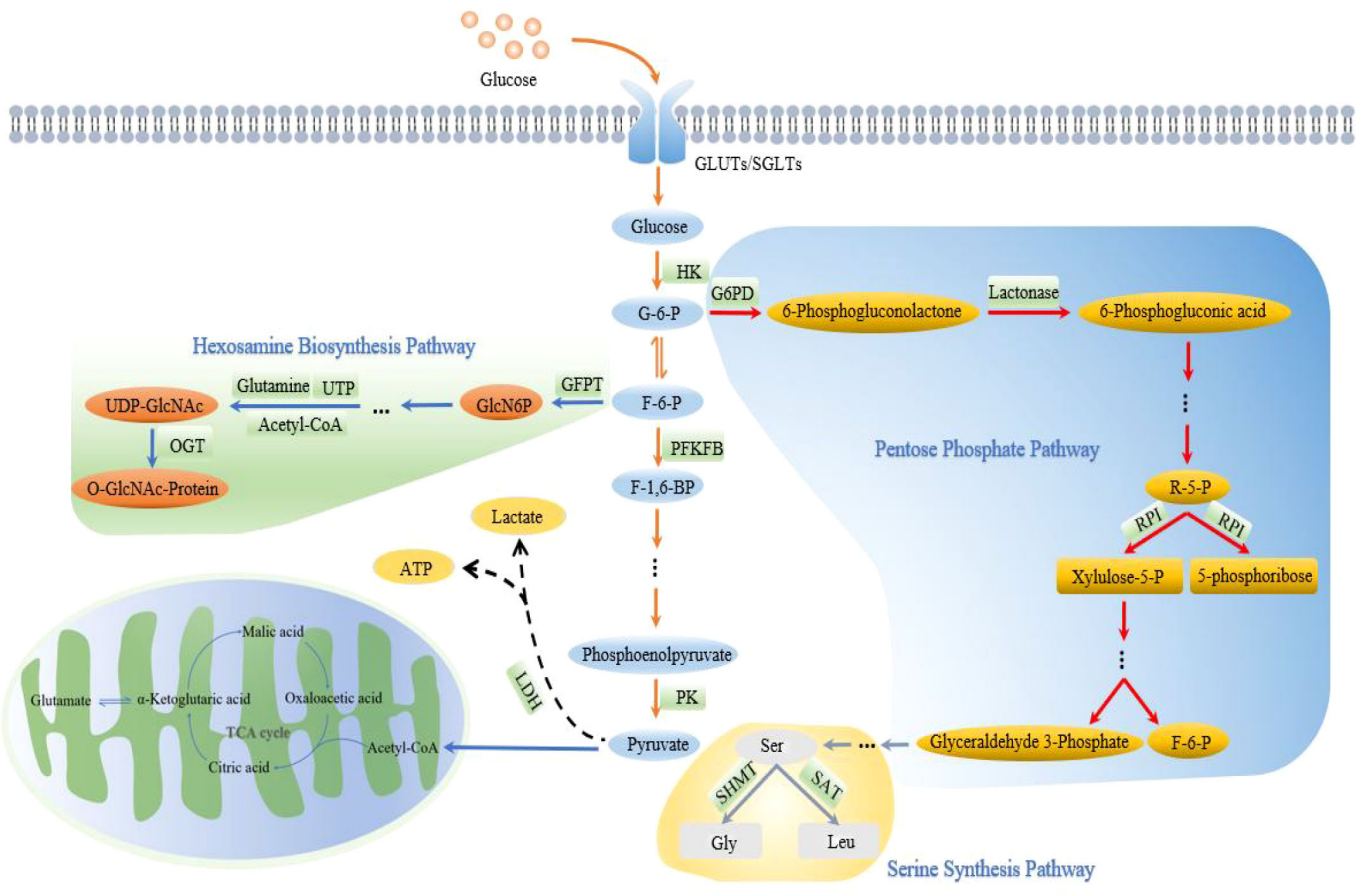
Aerobic glycolysis in PC. The entire process of glucose uptake and conversion and utilization through a series of pathways, including aerobic glycolysis pathway to generate lactate and ATP, PPP pathway to generate 5-phosphate ribose, serine pathway to generate Gly and Leu and hexosamine biosynthesis pathway to generate O-GlcNAc is shown.

Cancer progression is caused by factors beyond genetic changes, including metabolic alteration (13). Pfeiffer et al. revealed that glycolysis produces low ATP and high lactate compared with oxidative phosphorylation. Inefficient but high rate of ATP production favors unique growth advantages to cancer cells (14–16). At the same time, excessive lactic acid accumulation increases cancer cell resistance and immune evasion (17). These phenomena are prominently manifested in the lung (18), stomach (19), liver (20), breast (21), and prostate cancers (22). Metabolic reprogramming has also been observed in PC and has been linked to growth evasion, cell death resistance, rapid replication, angiogenesis, invasion, and metastasis of PC (23, 24). Aerobic glycolysis converts glucose to lactate and provides the carbon skeleton and the basis for the synthesis of macromolecular compounds (NAPDH and ATP) for the proliferation of PC cells (25). Aerobic glycolysis involves overexpression of glucose transporters, activation of key glycolytic enzymes, and increase of glycolytic flux, accumulation and transfer of glycolytic metabolic intermediates. **Figure 1** shows the aerobic glycolysis in PC cells.

### 2.1 Overexpression of glucose transporter

The transport of glucose transporters across the plasma membrane is the primary rate-limiting step in glycolysis. Glucose transporters on the cell membrane are mainly divided into facilitative glucose transporters (GLUTs), sodium-dependent active transporters (SGLTs) and newly discovered sugar transporters (SWEETs) (26, 27). GLUTs are passive transporters of glucose into cells along a concentration gradient. As one of the most important GLUTs, GLUT1 is a high-affinity glucose transporter linked to the malignant biological activity of tumours, particularly PC (28–31). Dysregulated GLUT1 expression promotes PC progression. Particularly, GLUT1 is not expressed on normal pancreatic acinar or ductal epithelium, but it is overexpressed in low-grade to high-grade PC lesions. Furthermore, GLUT1 is also closely related to the degree of malignancy degree, including tumour size, clinical stage, and lymph node metastasis. A meta-analysis of 538 PC patients found that GLUT1 expression positively correlates with tumour diameter (>2 cm) and lymph node metastasis (32). In addition, overexpression of GLUT1 in PC tissues correlates with that of of ki-67 (r=0.327; P=0.017) (33). The role of GLUT1 in PC suggests that it is a potential target for PC treatment under targeted therapy and neoadjuvant chemoradiotherapy, and shows favorable treatment response (34–36). Moreover, GLUT3 expression is also upregulated in PC. Studies have shown that hypoxia-inducing genes in PC cells include VEGF, GLUT1 and GLUT3, whose expression mediated by AMPK and HIF-1 activation. However, the mechanism by which GLUT3 promotes PC progression has not been clarified (37). The most studied members of the SGLT family are SGLT1 and SGLT2, which exerts diverse effects on PC. The expression of SGLT2 in tumour is higher than that of SGLT1. Despite low glucose concentration in the TME, SGLT2 can enhance glucose uptake by cancer cells. Meanwhile, inhibiting SGLT2 suppresses the proliferation of cancer cells, SGLT2, and not GLUTs, promotes PC progression (38, 39). Du et al. analyzed 88 included PC tissues and found that SGLT1 was overexpressed in pancreatic ductal adenocarcinoma (PDAC) and its expression correlated with overall survival (OS) and progression-free survival (PFS) of patients. Analysis of data in the Gene Expression Omnibus data base (GEO) and Oncomine databases have revealed comparable findings. However, no significant expression has been observed for SGLT2 (40). SWEETs, a novel glucose transporter encoded by SLC50, was first discovered in 2010 by Chen et al. (41). The SWEET1 subtype has recently been identified in the human genome, and is thought to promote cellular glucose efflux (42). Nevertheless, future studies in this area are needed.

### 2.2 Acceleration of glycolytic flux

Unlike oxidative phosphorylation, which produces abundant lactic acid and little ATP, this adaptation of tumour cells is not based on mitochondrial dysfunction, but is driven by the need for new biomass such as phospholipids, nucleotides, and amino acids. Therefore, entry of glucose enters in cells to accelerate glycolysis is critical for the growth and proliferation of tumour cells. For this reason, the three-step, almost irreversible rate-limiting link involving the key enzymes hexokinase, phosphofructokinase, and pyruvate kinase in the glycolytic process has become a key regulatory point of glycolytic flux. Hexokinase (HK), as a key enzyme that regulates the first irreversible phase of glycolysis, exists in four variants: HK1, HK2, HK3, and HK4. Compared with other subtypes, HK2 has a unique domain with 2 catalyzable structural sites, has no allosteric sites, and is more effective in promoting aerobic glycolysis of cancer cells (43–45). HK2 is overexpressed in PC cells. Overexpression of HK2 not only accelerates glucose uptake, but also activates multiple pathways required for glucose influx into Kras-driven PC growth, including the pentose phosphate pathway, serine synthesis pathway, and hexosamine biosynthesis pathway, which protomes the proliferation, invasion, and metastasis of PC (46, 47). This result can be achieved by modifying the pH of the TME and cell signaling factors (48, 49). The vascular endothelial growth factor A (VEGF-A) signaling pathway is one example, and its modifications influence PC angiogenesis and metastasis, which is associated with the increase in lactate accumulation driven by HK2 (50, 51). Phosphofructokinase (PFKFB) is a key regulator of glycolytic flux because it modulates the rate of fructose 1,6-bisphosphate production, consuming ATP (52). Among the four subtypes (PFKFB-1, PFKFB-2, PFKFB-3, and PFKFB-4) found in tumours, PFKFB-3 and PFKFB-4 are the most common in tumours (53–55). Chronic hypoxia and a nutrient-poor environment in PC increased the expression levels of PFKFB-3, PFKFB-4, and VEGF, all linked to the overexpression of hypoxia-inducible factor (HIF)-1α. Ultimately, these processes promote glycolytic flux and angiogenesis in cancer cells (56). Similar findings have been reported in gastric (56), breast (55), and lung cancers (57). Pyruvate kinase (PK), the final critical stage in glycolysis, determines the level of lactate accumulation. M2-PK, a pyruvate kinase in PC, is regulated by fructose-1,6-bisphosphate and alanine allosteric and participates in the production of pyruvate. This generates abundant lactate under the action of lactate dehydrogenase, achieved under hypoxia or aerobic conditions. Consequently these processes raw materials for the production of nucleotides and amino acids in oxygen-containing cancer cells (58). Li and colleagues verified the regulatory role of M2-PK (59). While glycolysis efficiently provides energy for tumour growth, the large amount of lactate produced can also serve as an intermediate metabolic substrate for the tricarboxylic acid cycle. However, in pancreatic tumours, this substrate is more derived from the contribution of glutamine, which is worth further exploration in the future (60).

### 2.3 Accumulation and transfer of metabolic intermediates

Tumour growth not only requires abundant lactates produced by glycolysis, but also requires pathways such as the pentose phosphate pathway (PPP), serine pathway, and hexosamine biosynthesis pathway, which provide macromolecular substrates for the phospholipids, nucleotides and amino acids required for cancer cell proliferation. The PPP, critical in glycolysis, has been implicated in key regulatory processes of biosynthesis and redox reactions. Unlike glycolysis and oxidative phosphorylation, PPP does not produce ATP, but rather NADPH and 5-phosphate ribose *via* oxidative and non-oxidative processes, respectively (61). NADPH play a role in the synthesis of reduced glutathione and maintains cell redox state, whereas ribose 5-phosphate constitutes the sugar of purine and pyrimidine nucleotides (62). Furthermore, ribose-5-phosphate isomerase (RPI) links the PPP oxidative pathway with the non-oxidative pathway to generate more ribose 5-phosphate, both of which provide carbon for *de novo* synthesis of nucleotides and non-essential amino acids skeleton synthesis (63). Ying and colleagues demonstrated the mechanism that drives non-oxidative respiration in PC is more defined than oxidative respiration (47). Non-oxidative PPP promotes the proliferation and invasion of PC and induces gemcitabine resistance. This mechanism is intimately related to the massive accumulation of metabolic intermediates 5-ribose phosphate, fructose 6-phosphate (F6P), and glyceraldehyde 3-phosphate (G3P) and involves transketolase (TKT), ribose 5-phosphate isomerase (RPI), and ribulose 5-phosphate epimerase (RPE) (47, 64, 65). As another key bypass of glycolysis, serine is metabolized by 3-phosphoglycerate through a series of enzymatic reactions, such as dehydrogenation of 3-phosphoglycerate dehydrogenase (PHGDH), catalytic transamination of phosphoserine aminotransferase (PSAT), and catalytic hydrolysis of phosphoserine phosphatase (PSPH). Degradation of serine-by-serine hydroxylmethylase (SHMT) provides important precursors for macromolecular biosynthesis, such as glycine, which accelerates the proliferation of cancer cells. Furthermore, glycine synthase breaks down the accumulated glycine, providing a carbon source for the one-carbon metabolic pathway. In addition, the glycine cleavage system (GCS) as well as choline and other amino acid metabolic pathways, are sources of single carbon (66). The enhancement of one-carbon metabolism with the folic acid cycle and methionine cycle as the core promotes nucleotide synthesis, lipid metabolism, and redox state maintenance of cancer cells (67). Therapies that target the single-carbon metabolic pathway, such as serine hydroxymethyltransferases (SHMT1 and SHMT2) inhibition, the key enzymes that convert serine to glycine, and lowering entry of monocarbons to the tetrahydrofolate (THF) cycle, are promising for anti-cancer therapies. In addition, limiting dietary intake and increasing serine synthesis have emerged as a new direction for PC, which is particularly for PC cells lacking live kinase B1 (LKB1) (68). Recent studies have demonstrated that phosphoglycerate dehydrogenase (PHGDH) is critical essential in the growth of serine-deprived PC cells, compensates for the lack of serine and glycine synthesis, and promotes the proliferation and metabolic activity of PC. However, regulating the methylation of the PHGDH promoter region is particularly critical for PHGDH activation (69). Glutamine, acetyl-CoA, and UTP, participate in the catalysis of glucose 6-phosphate generated from the glycolytic pathway by several enzymes to generate UDP-GlcNAc, which becomes O-linked N-acetylglucosamine (O-GlcNAc) modified substrate. The hexosamine biosynthesis pathway (HBP) mediates glucose metabolism, lipid metabolism, and amino acid metabolism, and participates in gene transcription, cancer cells signaling, transduction, and proteolysis, among other activities (70). HBP provides sufficient glutamine and growth factor signals for tumour growth and survival by mediating glucose and glutamine metabolism, particularly when glucose is depleted (71). Related studies have shown that Kras-mediated PC cells caused exhibit high glycolytic activity, and transfer of glucose to PPP and HBP for metabolism under normoxic conditions. This enhances glutamine synthesis, providing providing substrates for glycosylation reactions and promoting the proliferation rate of PC cells. At the same time, there is an increase in glycolytic activity in the hypoxic areas of pancreatic tissue, and the excess lactate produced provides glucose rather than carbon source for oxygenated cancer cells (72). **Table 1** summarizes the mechanism and clinical relevance of key enzymes in the glycolysis process that promotes the progression of PC.

**Table 1:** Summary of the mechanism and clinical relevance of key glycolytic enzymes in the progression of PC.

Glycolytic enzymes	Mechanism of action	Clinically relevant	Related inhibitors	References
GLUT1	Promotes glucose uptake by PC cells	Progression, clinical staging, prognosis	Glutipyran, Apigenin	(32–36)
GLUT3	Promotes glucose uptake by PC cells	Progression	Glycogen synthase kinase-3β inhibitors	(37)
SGLT1	Promotes glucose uptake by PC cells	Progression, prognosis	Phlorizin	(38, 39)
SGLT2	Promotes glucose uptake by PC cells	Progression	Canagliflozin	(40)
HK2	Promotes glucose uptake by PC cells, activates the pentyl phosphate pathway, serine synthesis pathway, and hexosamine biosynthesis pathway	Progression	Lonidamine, Everolimus	(46, 47)
PFKFB3/4	Consumes ATP and regulates the rate of fructose production of 1,6-bisphosphate	Progression	Aurintricarboxylic acid	(56)
M2-PK	Participates in the production of pyruvate and produces a large amount of lactic acid under the influence of lactate dehydrogenase	Progression	L-phospholactate, M2-PK-binding peptide aptamers	(58, 59)
RPI	Promotes the production of ribose 5-phosphate, providing raw materials for the synthesis of purines and pyrimidines	Progression, prognosis	SRC-2	(63)
SHMT	Promotes glycine synthesis and provides raw materials for one-carbon metabolism	Progression	5-substituted pyrrolo[3,2-d]pyrimidine compounds	(66)
PHGDH	Catalyzes the synthesis of serine	Progression	Ixocarpalactone A	(66, 69)
GFPT	Promotes hexosamine biosynthetic pathway and provides substrates for glycosylation reactions	Progression	6-diazo-5-oxo-l-norleucine [DON]	(70–72)

## 3 Crosstalk between TME and glycolysis in pancreatic cancer

**Figure 2:**
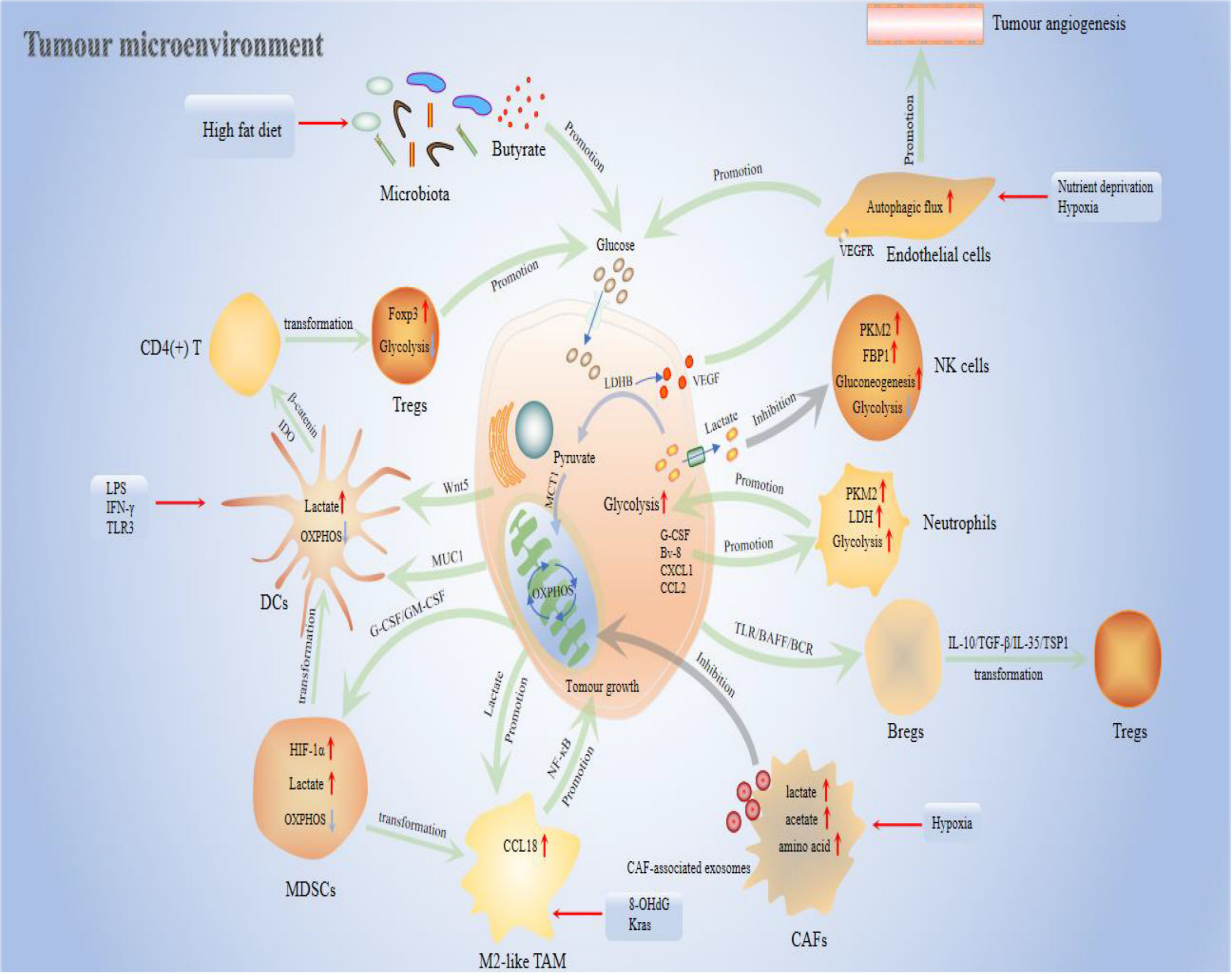
Metabolic crosstalk between PC cells and the TME. The figure completely shows the roles and pathways of important components in the TME in the glycolytic process, proliferation, and invasion of PC, including CAFs, TAMs, DCs, neutrophils, NK cells, Tregs, Bregs, endothelial cells, MDSCs, and microbiota.

In tumour progression, the surrounding extracellular matrix (ECM) undergoes active dynamic remodeling, including massive fibronectin and collagen deposition, linear arrangement of collagen fibers, and increased stiffness. This is besides the continuous activation of fibroblasts, immune infiltration, and angiogenesis, constituting the internal environment that distinguishes normal tissue survival, that is, TME (73). The presence of TME provides a basic barrier for tumour immune escape, deterioration, and promotes aggressiveness, and chemotherapy resistance. However, metabolism is essential to this process, specifically glycolysis in tumour cells. From normal pancreatic epithelium to dysplasia and the evolution of malignant tumours, environmental factors, including hypoxia, oxidative stress, and acidosis promote metabolic programming in PC cells, especially the enhancement of glycolysis. This result reversely reshapes the microenvironment in the direction of tumour growth through vicious circle (74–76). This review summarized inextricable links between the TME and PC glycolysis, and the metabolic changes that promote the growth of PC. **Figure 2** shows metabolic crosstalk between PC cells and TME.

### 3.1 Fibroblasts

Cancer-associated fibroblasts (CAFs) are the most abundant components in tumour stroma, affecting tumour angiogenesis, signal transduction, metabolism, immunosuppression, and chemotherapy resistance by secreting various growth factors, inflammatory mediators, and matrix proteins. This is inseparable from the molecular and functional heterogeneity of CAFs, which depend on various internal and external stimuli to stimulate precursor CAFs to produce different phenotypic features, including myCAF, iCAF, apCAF, and release-related signals, ultimately affecting tumours development (77–81). In the TME of chronic hypoxia and nutritional deficiency, exosomes secreted by CAF carry significant lactate, acetate, and amino acids to promote tumour metabolism, including PPP, glutamine decomposition, and nucleotide metabolism. Moreover, CAF-secreted exosomes significantly reduced the membrane potential of mitochondria in cancer cells, affecting its normal function of mitochondria as well as reducing the level of their oxidative phosphorylation, accompanied by a compensatory increase in tumour cell glycolysis. This is closely related to the high glucose uptake and lactate secretion of cancer cells by exosomes, ultimately inducing the Warburg phenotype (81, 82). Previous studies have confirmed that the mitochondrial reserve capacity of PC cells reduces in the presence of exosomes, suggestive of inhibited mitochondrial function by CAF-derived exosomes (83, 84). The above study is based on the oxidative phosphorylation inhibition of mitochondria, which promotes the proliferation tumour cells by enhancing glycolysis. However, metabolic rearrangement increases the dependence of cancer cells on other nutrients. The enhancement of the glutamine metabolic reduction pathway provides acetyl-CoA for the growth of cancer cells, partially explaining the source of plasma membrane synthesis raw materials (fatty acid salts) for PC cells when glucose supply is insufficient, and exosome-mediated upregulation is independent of the effect of Kras (83, 85–87). Nevertheless, the important role of exosomes as a bridge of communication between CAFs and PC cells remains underexplored. Among the PC subtypes, the mesenchymal-like subtype, primarily PANC-1 and MIA PaCa-2 cell lines, exhibits enhanced aerobic glycolytic activity and reduces matrix, collagen and activated PSC content by secreting CSF-1, which is beneficial to reduce the mechanical limitation in the tumour matrix and is associated with a worse clinical prognosis. In contrast, the epithelioid subtype, primarily comprises Capan-2 and AsPC-1 cell lines, rich in matrix content. The use of glucose is oriented towards the TCA cycle and fat production (88, 89). The effect of CAF on the metabolism of different subtypes of PC needs further exploration to identify novel clinical therapeutic targets.

### 3.2 Endothelial cells

Vascular endothelial cells adjacent to or distant tumour tissue and bone marrow-derived endothelial progenitor cells, are activated by several regulators to form abnormal vascular mosaicism. This provides continuous nutrition for the growth and metastasis of cancer cells, including amino acids and phosphates (90, 91). These activities depend on high endothelial autophagic flux and are sustained by the nutrient-deprived, hypoxic TME, providing continuous stimulation (92–94). Furthermore, tumour cells require a continuous supply of several new blood vessels for more nutrients. This explains, to a certain extent, the richer blood supply of cancer cells than normal cells. Of note, the proliferation of endothelial cells is fundamental in the above process. High autophagy is a metabolic strategy resolving nutrient starvation, particularly in the face of persistent oxidative stress (95, 96). At this time, the glycolytic rate of tumour cells in the hypoxic area increases, producing significant lactate, which is then converted into pyruvate by the oxidation of MCT1 and LDHB oxidation and accelerates oxidative phosphorylation of tumour cells in the oxygen-containing region, which realizes the metabolic symbiosis of hypoxic and oxygen-containing cancer cells. Consequently, oxidized cancer cells prefer lactic acid over glucose to sustain their growth and proliferation (97–99). During this process, LDHB activation induces V-ATPase dependent lysosomal acidification and autophagy, which activates the Janus kinase 2 (JAK2)/signal transducer and activator of transcription 3 (STAT3) pathway. These events promote VEGF secretion, which upregulates the secretion of vascular endothelial growth factor receptor (VEGFR), and promotes the proliferation of endothelial cells and angiogenesis (95, 100–103). For PC with dense interstitial density, although it is in a chronic hypoxic environment, aerobic glycolysis promotes invasion, metastasis, and epithelial-mesenchymal transformation *via* the Hif-2α signaling pathway. Nevertheless, the specific mechanism remains unclear and needs further investigation (104).

### 3.3 Immune cells

The metabolic competition between immune and tumour cells in the microenvironment causes immunosuppression, mediated by the enhanced glycolytic activity of tumour cells, which in turn augments the proliferation of malignant tumours. Therefore, immunosuppression is an important indicator of tumor malignancy degree (24, 105, 106). The infiltration degree, phenotype ratio, and substance secretion of immune cells promote cancer cells proliferation. For nutrient-deficient PC cells, the high demand for nutrients alters the metabolic activities of peripheral immune cells, ultimately providing an environment for tumour immune escape (107, 108). We systematically summarize the relationship between high glycolytic activity and immune cells in PC.

#### 3.3.1 T lymphocytes

Under the stimulation of inflammation, viral infection, tumour, and other factors, pluripotent stem cells derived from bone marrow differentiate into different types of T lymphocytes, which perform corresponding functions. For instance, helper T cells [CD4(+) T] kill specific cells and mediate humoral and cellular immunity. In the process of tumour growth, specifically for PC, CD4(+) T cells differentiate into regulatory T (Treg) cells due to the demand for abundant energy demands and biological raw materials. Ideally, tumour-derived Wnt5 signaling induces activates β-catenin in tumour-associated DCs (TADC), causing secretion of indoleamine 2,3-dioxygenase (IDO), which promotes the generation of Treg cells to suppress anti-tumour T cell activity (109, 110). Since Treg cells are not glucose-dependent, they express low levels of GLUT1, and inhibit Myc and glycolysis *via* the transcription factor Foxp3, improve oxidative phosphorylation and increase the NAD: NADH ratio to survive in a low glucose and high lactic acid environment (specifically the PC microenvironment). Consequently, this metabolic competition with tumour cells. In contrast with glucose-dependent cytotoxic and effector T cells (high expression of GLUT3), the mechanisms by which Treg cells metabolize increases their survival in TME. Additionally, Treg cells can provide energy for their own development by absorbing and using lactic acid in TME. Treg cells also provide positive feedback to regulate the aerobic and glycolytic process of cancer cells, thereby promoting tumour immune escape and proliferation (111–115). This partly explains the shorter disease-free survival and overall survival of cancer patients with high Treg cells infiltration (116, 117). Even when stimulated by oxidative stress signals in TME, Treg cells also do not undergo apoptosis. The above behavior of Treg cells depends on the regulation of GPX4 regulation (ferroptosis pathway regulation and production), which neutralizes lipid peroxidation and prevents the formation of ferritinase, and maintains the activation of Treg cell activation thereby promoting anti-tumour immunity and cancer progression (118, 119). Subgroup analysis of PC metabolism revealed low glycolytic subtypes, including quasi-mesenchymal immunoinfiltration, especially for CD4+ T cells. This is mainly caused by excessive lactic acid production in the microenvironment caused by the of MCT4 and LDHA upregulation in the above isoforms (120). Metabolic inhibition of Tregs and improvement of the metabolic competitiveness of effector T cells are critical to improving anti-tumour immunity.

#### 3.3.2 Macrophages

Macrophages are important cells that maintain tissues and immune systems. In the PC stroma, macrophages are the most abundant infiltrating immune cells, which can interact with interstitial cells, including cancer cells and adipocytes, to regulate metabolism, inflammation, and immune status, ultimately forming an immunosuppressive TME (121). The study found that F4/80-labeled TAMs had higher glucose affinity and metabolic rates than infiltrating T cells and cancer cells (122). Under the influence of nutritional deficiency, specific transcription factors, hypoxia, oncogenes, and other factors, PC cells metabolically change from oxidative phosphorylation to glycolysis, further inducing phenotypic changes of TAM (transition to M2 TAM); that is glycolysis of PC cells to produce substantial lactate (123). This was elaborated by Ye et al., who believed that a colossal amount of lactate produced by cancer cells through glycolysis causes the polarization of TAM to M2 phenotype in a highly infiltrated PC microenvironment by macrophages. Subsequent M2-type TAMs secrete large amounts of several C-C motif chemokine ligand 18 (CCL18) and activate vascular cellular adhesion molecule-1 (VCAM-1) in cancer cells *via* noncanonical nuclear factor-κB (NF-κB) signaling to enhance the Warburg effect, which forms a positive feedback loop. The M2-type TAM also relies on glycolysis to further promote the vascular extravasation of cancer cells, inducing tumour epithelial-mesenchymal transition (EMT) and transforming growth factor beta (TGF-β)-dependent distant dissemination (124–126). However, the mechanisms by which VCAM-1 affects the glycolysis process and glucose uptake and lactate production of tumour cells remain to be explored. Moreover, for PC subtypes with high expression of MCT4, such as the quasi-mesenchymal type, excessive lactic acid production is conducive to macrophages biased towards anti-inflammatory and cancer-promoting M2 transformation. However, while infiltrating M2 macrophages promote epithelial-mesenchymal transformation and enhance drug resistance in PC, their high HLA-DR expression also affects the composition of other cell populations in the microenvironment, including CD4+ T cells and MDSCs. However, this mechanism needs to be further studied. The upregulation of MCT4 levels also indicates a poor prognosis for this subset, providing novel new ideas for developing immune and targeted metabolic therapies for PC (127).

#### 3.3.3 Natural killer cells

As an important cell population for cancer immune surveillance, natural killer (NK) cells are highly dependent on glucose-mediated glycolysis, and activated NK cells utilize high glycolysis rates to support their own rapid proliferation. Tumour-induced glucose restriction in the TME reduces NK cell glycolysis and disrupts normal NK cell effector functions, such as interferon-gamma (IFN-γ) production and target cell damage, ultimately suppressing antitumour capacity (128–132). NK cell metabolism is dependent on the efficient regulation of the pyruvate kinase (PK), particularly the PKM2 isoform, which is preferentially expressed in NK cells and enables them to rapidly regulate glycolytic flux and maintain anabolism or catabolism. However, it cannot sufficiently maintain the normal activity of NK cells in the TME, because PKM2 is also highly expressed in many cells with high biosynthetic load, especially tumour cells, including PC (133–135). Moerover, tumour cells produce large amounts of fructose 1,6-bisphosphatase 1 (FBP1), a critical enzyme involved in gluconeogenesis in NK cells, which limits the mutually exclusive process of glycolysis process and thereby decreasing NK cytotoxicity and phenotypic dysfunction (136). In addition, the acidic environment created by the huge amount of lactic acid produced by tumour cells decreases the activity of NK cells, ultimately inhibiting the anti-tumour effect (137). The regulation of NK cell metabolism mediates the beneficial effects of anti-cancer therapy in PC patients.

#### 3.3.4 Dendritic cells

As is with macrophages, dendritic cells (DCs) can rapidly respond to invading pathogens and they constitute the body’s early defense barrier. Since DCs act as antigen-presenting cells for T cell activation that drive anticancer effects, tumour survival requires the disruption of normal DCs functions, including their maturation and metabolism. For instance, mucin 1 (MUC1) from PC cell lines can inhibit DC differentiation and maturation, impairing their abilities to effectively activate T cell (138). In the TME of nutrient deficiency and hypoxia stimuli including lipopolysaccharide (LPS), IFN-γ, and toll-like receptor 3 (TLR3) ligands can induce a metabolic switch in DCs from oxidative phosphorylation to glycolysis. This metabolic reprogramming allows DCs to adapt to this environment and to exert their anti-cancer effects. Elevated glucose uptake levels by DC cells with high CD103 expression is vital for maintenance of CD8+ T cell activities (139). However, tumour-mediated glucose deprivation and the large amounts of lactate lactate inhibits DC differentiation and maturation, reducing glucose uptake as well as utilization, and inhibit protein glycosylation in DC cell ER, resulting in accumulation of unfolded proteins in the ER lumen to induce immunosuppressive ER stress responses (140–143). In addition, changes in the mechanical tension of tumour cells affect the activation and flux of glycolytic pathways in DC cells, which involves the key regulation effects of TAZ and PIEZO1, but the specific mechanisms should be conclusively determined (144). Moreover, activation of the aberrant Wnt signaling pathway has been implicated in immune evasion. The Wnt ligands secreted by tumour cells activate the transformation of CD4(+) T cells to Treg cells by promoting the expression of β-catenin expressions in DCs, reducing the burden on tumour metabolic burden (145, 146). In summary, targeting DC metabolism may help enhance anti-tumour effects.

#### 3.3.5 Myeloid-derived suppressor cells

The bone marrow-derived heterogeneous cell populations with immunosuppressive functions are produced in large quantities under the stimulation of various factors, including cancer and inflammation. This cell populations are referred to as myeloid-derived suppressor cells (MDSCs), which are mainly grouped into monocyte population (M-MDSCs) and granulocyte population (G-MDSCs) (147). In the TME, MDSCs inhibits T cells activation, reduce NK cells cytotoxicity, facilitate Treg cells development and prevent DC cells maturation to accelerate tumour progression (148, 149). In a triple-negative breast cancer model, tumour cells coordinate AMP-activated protein kinase (AMPK)-ULK1, autophagy, and CCAAT/enhancer-binding protein beta (CEBPB) molecular network *via* aerobic glycolysis to promote the secretion of granulocyte colony-stimulating factor (G-CSF) and granulocyte macrophage colony-stimulating factor (GM-CSF), and to maintain the growth and development of MDSCs to evade tumour immunity (150). Immature MDSCs are also highly dependent on glycolysis. Their metabolic transition from oxidative phosphorylation to glycolysis is dependent on the transduction of phosphatidylinositol 3-kinase (PI3K)-AKT-mammalian target of rapamycin (mTOR) signaling, which then subsequently activates HIF-1α, resulting in increased expressions of GLUT1, HK2/3 and lactate transporters, key enzymes of glycolysis, thereby promoting the glycolytic flux. This activity is particularly pronounced for MDSCs with elevated CD11b (151–153). Unlike other immune cells, MDSCs can be induced to differentiate into M2 TAMs or DCs in the TME, and their metabolite (phosphoenol pyruvate) suppresses ROS overproduction, promotes the survival of MDSCs, and reduces apoptosis. Therefore, MDSCs can effectively promote cancer cell proliferation. Moreover, the high glucose uptake rate of MDSCs effectively reduces the glucose availability for anti-cancer immune cells, leading to their dysfunctions and apoptosis. In contrast, tumour metabolic activities can be achieved *via* glutamine uptake and utilization, which ultimately promotes tumour growth (154). However, in different TMEs, especially in PC microenvironment, the effects of proportional and metabolic differences between MDSCs subsets on tumour cells as well as the effects of high glucose metabolism rate of MDSCs and their markers on the clinical prognosis of PC patients remain to be elucidated.

#### 3.3.6 Neutrophils

As the first line of defense against the invasion of external pathogens, neutrophils rapidly increase in abundance and make an effective immune response during inflammation, injury and tumours development. Neutrophils have strong plasticity, especially in tumours, breaking the long-standing concept of the inherent phenotype and function of neutrophils. Fridlender et al. showed that tumour-associated neutrophils can switch from anti-tumour to tumour-promoting phenotypes, which revealed the functional diversity and heterogeneity of neutrophils (155). The continuous secretion of recruitment factors [such as G-CSF, Bv8, C-X-C motif ligand 1 (CXCL1) and CCL2] by tumour cells into the microenvironment enhances neutrophil infiltration and stimulation of the microenvironment enhances the activities of lactate dehydrogenase and PKM2 in neutrophils, which in turn uses anaerobic glycolysis to generate energy. However, metabolic coordination between cancer cells and neutrophils has yet to be conclusively determined (156, 157). Notably, neutrophil subsets are transformed towards tumour promotion. For instance, the newly identified C5aR1 subset, whose phenotypic markers include CD66B, CD15, and C5aR1, can be transferred *via* extracellular signal-regulated kinase 1/2 (ERK1/2)-WTAPENO1 signaling to promote the glycolytic capacity of breast cancer, including the rate of glucose uptake and lactate production. There is a need to determine whether this subpopulation exists in other tumours, including PC. In summary, enhancement of tumor metabolism can be achieved by affecting neutrophil phenotypes and functions (158).

#### 3.3.7 B lymphocytes

Under stimulation by exogenous antigens, hematopoietic stem cells are induced to mature into B lymphocytes, which will then secrete various pro-inflammatory and anti-inflammatory cytokines to play a variety of immune regulation functions in the body (159). However, due to high plasticity, B lymphocytes are often induced to differentiated into different subpopulations by environmental stimuli. The most prominently expressed B cells in the TME are the regulatory B cells (Bregs) that produce immunosuppressive factors. Bregs prevent immune-mediated inflammatory tissue damage and inhibits the immune surveillance mechanisms that prevent cancer development and viral infections. These immunosuppressive factors include interleukin (IL)-10, TGF-β, IL-35 and thrombospondin 1 (TSP1) (159–161). In the TME, stimulated by signals such as TLR, B cell activating factor (BAFF), and B cell receptor (BCR), the glycolytic flux of Bregs increases, and is highly infiltrated in the TME. This results in secretion of a large number of immunosuppressive factors, inhibiting cytotoxic T cells function and inducing their transformation into Tregs, which promotes the acceleration of the glycolytic process in tumour cells (162, 163). Studies on the relationship between B lymphocytes and tumours, especially PC, have majorly focused on direct or indirect molecular targets, such as activation of STAT3 to promote tumour angiogenesis (164). There is a need to elucidate on how tumour cells metabolically affect the directional differentiation of B lymphocytes and how Bregs establish associations with other immune cells in the TME to affect tumour cell metabolism and proliferation. **Table 2** summarizes immune cell metabolism patterns in the TME and their effects on PC.

**Table 2:** Immune cell metabolism patterns in the TME and their effects on PC.

Component	Metabolic mode	Key regulators	Clinical significance	References
Treg cells	Oxidative phosphorylation, lipid metabolism	Foxp3	Promote glycolysis of PC and promote immune escape, which is associated with poor prognosis of patients	(111–115)
CD8+ T cells	Glycolysis, oxidative phosphorylation	GLUT3	Promotes anti-tumour immunity	(111–115)
M1 macrophages	Glycolysis, pentose phosphate pathway	MCTs	Good for antitumour immunity	(124–126)
M2 macrophages	Oxidative phosphorylation, lipid metabolism	MCTs	Promotes glycolysis in PC cells and tumour progression	(124–126)
NK cells	Glycolysis	PKM2, FBP1	Anti-tumour effects	(133–135)
DCs	Glycolysis	MCT, TAZ, PIEZO1	Anti-tumour effects	(139–144)
MDSCs	Glycolysis	GLUT1, GLUT3, HK2/3 MCTs	Facilitates immune escape	(151–154)
Neutrophils	Glycolysis	LDH, PKM2	Promotes glycolysis	(156, 157)
Breg cells	Glycolysis	TLR, BAFF, BCR	Promotes glycolysis and facilitates immune escape	(162, 163)

### 3.4 Microbiota

Rapid advances in sequencing technology have promoted studies on macroscopic roles of gut microbiota, which is important for some cancers that are difficult to be diagnosed early, treated and evaluated. Gut microbiota disorders and imbalances can often lead to development of gastrointestinal tumours, for instance, *Helicobacter pylori* is closely associated with esophageal cancer and gastric cancer (165, 166); *Actinobacteria, Proteus and Clostridium spores* are associated with PC (167, 168), while *Escherichia coli and Bacteroides fragilis* are associated with colorectal cancer (169). Initially, it had been postulated that microbial changes have insignificant effects on tumour susceptibility, especially PC, and that tweaking the microbiome structure or composition does not affect the poor prognosis of PC patients (170). However, with advances in detection technologies, it has been found that microorganisms are closely associated with the occurrence, development and treatment of PDACs. *Porphyromonas gingivalis (P. gingivalis), Fusobacterium, Neisseria elongata (N. elongata) and Streptococcus mitis (S. mitis)*, which cause oral microbial flora imbalances, are the key pathogenic bacteria involved in pancreatic carcinogenesis (171). *Helicobacter pylori*, which is closely related to gastric mucosal lesions, has also been associated with chronic pancreatitis or PC development. There is a need to determine whether it can cause an increased the risk of PC development (172–175). Moreover, the mechanisms of action of HBV and *Escherichia coli* in PC should be explored further (176–178). Microbiota imbalances activate the inflammatory responses in the pancreas, leading to the accumulation of inflammatory cells and pro-inflammatory factors. Long-term inflammation is accompanied by a persistent oxidative stress state, resulting in metabolic changes in cells, imbalanced abundant of tumour-promoting and tumour-suppressing factors as well as tumour occurrence and development of tumours (178). Modulation of diet or energy intake is effective in correcting microbiota imbalances, but has minimal effects in cancer treatment. However, microbiome modulation may be crucial in immunotherapy. Phenotypic remodeling of immune cells in the TME by microbes enhances or weakens tumor immune resistance, thus, the roles of the microbiota are two-sided. The above conclusions are limited to the regulation of targeted immune cell balance, and studies should determine whether microbial regulation can cure PC (179–181). Microbial metabolism affects systemic homeostasis and remodels the TME *via* metabolites, affecting tumour angiogenesis, metabolism, and EMT (182, 183). Microbial dysbiosis in obese cancer patients has a greater impact on cancer, including PC, and is closely associated with tumour metabolism (184, 185). Regardless of duration, a high-fat diet, which is closely linked to obesity, can significantly alter the composition of gut microbes. For example, a high-fat diet causes down-regulates *Lactobacillus enterica*, which subsequently leads to decreased expressions of sodium-glucose co-transporter and glucagon-like peptide 1 (GLP1), resulting in insulin resistance and elevated blood glucose levels as well as activation of oncogenic KRAS and induction of aerobic glycolysis. These outcomes promote PC progression (186, 187). Short-chain fatty acids, which are produced by decomposition of dietary fibers by intestinal commensal bacteria, have inhibitory effects on colorectal and breast cancers (188–190). The low levels of acetate, propionate and butyrate, which synthesize short-chain fatty acids in cancer, reduce the inhibition of tumour cells, which is partly associated with the regulation of the activity of cancer cell mitochondria and the promotion of energy expenditure. Geng et al. reported that butyrate, a synthetic raw material for short-chain fatty acids, inhibits the glycolytic rate of cancer cells by suppressing the concentrations of GPR109a-AKT signaling-mediated membrane (GLUT1) and cytoplasmic glucose-6-phosphate dehydrogenase (G6PD), thereby exerting anti-cancer effects. These gut commensal bacteria include Bacteroides, Bifidobacterium, fungi, Streptococcus, Lactobacillus and Enterobacter (191). But there is a need to establish whether PC cells also inhibit the production of short-chain fatty acids by microbiota to promote their own progression remains to be further investigated. In addition, it is worth paying attention to whether bacteriocins such as nisin, which are produced by *lactic acid bacteria* and *Escherichia coli* have comparable anticancer effects in PC. In summary, in addition to regulating the immune cells in TME to influence the progression of PC, the microbiota plays an important role in PC cell glycolysis. However, studies should elucidate on the proportion of dominant bacteria and exploration of metabolic mechanisms.

## 4 Glycolysis in pancreatic cancer and lactic acidification modification

Metabolic changes that involve the transfer of glucose utilization to increase lactic acid production have become a fundamental hallmark of tumours (192). Increased lactic acid accumulation induces acidification of the microenvironment, which also involves processes such as glutamine metabolism. This promotes tumour proliferation, invasion and metastasis, angiogenesis, as well as drug resistance (193). Moreover, lactic acid stimulation and the acidic TME affect the cells in the microenvironment to varying degrees, which is conducive for tumour immune escape (194). These effects are based on changes in lactate levels and participation in metabolic activities. However, lactic acid can also participate in cellular metabolic activities, as an epigenetic modification substrate. For the first time, Zhao Yingming et al. confirmed that histone H3K18 lactic acidification modification is involved in macrophage polarization, which provides an important reference for exploration of lactic acid modification mechanisms in cancer progression (195). Histone lactic acidification can promote tumour development, for instance, it promotes ocular melanoma by activating YTHDF2 expressions, and can also affect the malignant phenotypes of non-small cell lung cancer by regulating the expressions of key enzymes in glycolysis and TCA (196, 197). Histone lactic acidification is also involved in progression of liver, breast and colon cancers (195). However, the effects of histone lactic acidification on glucose metabolism and PC progression have yet to be established, but its effects on TAM in the TME are particularly obvious. As earlier mentioned, production of large amounts of lactic acid can induce TAM transformation to the M2 type in the microenvironment, leading to malignant progression, which also involves the modification of histone lactic acidification. When tumour cells invade, macrophages are polarized into the M1 or M2 phenotypes, however, over time, the increased accumulation of TME lactate levels increases lactate levels and histone lactation levels in M1 macrophages, which induces the expressions of M2-like phenotypes (Arg1 and VEGFA). Histone lactation acts as a “lactate timer”, using epigenetic modification mechanisms to induce M2-like characterization (195, 198). Studies should elucidate on epigenetic modification mechanisms of histone lactic acidification in PC.

## 5 Glycolysis in pancreatic cancer and ferroptosis

**Figure 3:**
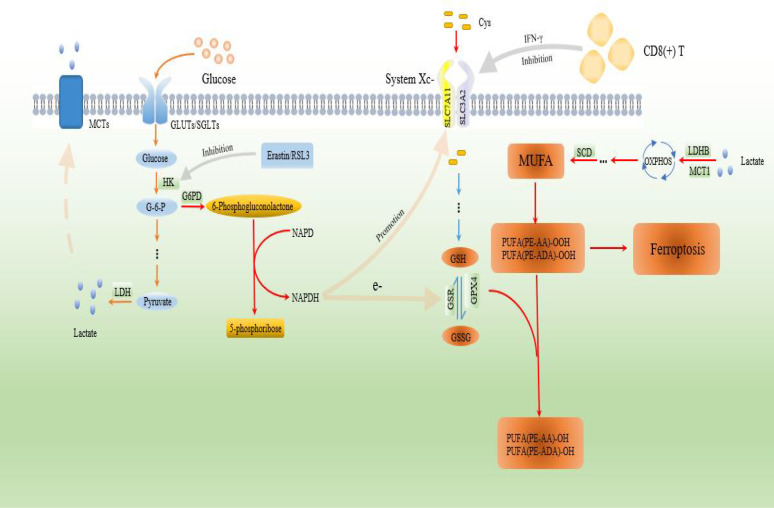
The association between PC glycolysis and ferroptosis and TME. The NAPDH that is produced by the PPP pathway in PC cells provides sufficient reducing equivalents for the synthesis of GSH. In addition, a large amount of lactate produced by the glycolytic pathway in cancer cells and TME undergoes a series of enzymatic reactions to generate MUFA, which reduces ferroptosis occurrence. However, the IFN-γ derived from CD8(+) T cells in the TME induces ferroptosis by inhibiting system Xc-, thereby suppressing malignant progression of PC.

Ferroptosis, which results from interactions of iron aggregation, lipid reactive oxygen species production, and glutathione-dependent decline in GPX4 activities, provides new insights into forms of cell death that are distinct from autophagy, apoptosis, and necrosis (109). Perhaps, due to rapid growth, tumour cells require need a lot of energy generated by oxidative phosphorylation, but they are inherently more likely to lead to enhanced reactive oxygen species (ROS) producing, resulting in the occurrence of ferroptosis, which is detrimental to their own growth. Therefore, tumour cells are more inclined to use the high-rate form of glycolysis to alleviate the unfavorable situation caused by ROS stress. For instance, erastin and Ras-selective lethal small molecule 3 (RSL3) results in tumour cells ferroptosis and a decrease in activities of the key rate-limiting enzymes (HK2 and PKM2) in glycolysis (199, 200). Enhanced glycolysis in tumour cells is often accompanied by an increase in PPP activities (64, 201). The glycolytic intermediate metabolite (G6P) synthesizes ribose 5-phosphate, erythrose 4-phosphate and NAPDH *via* oxidative and non-oxidative branched pathways in the PPP (202). During the reduction of glutathione disulfide (GSSG) to glutathione (GSH), NAPDH acts as an electron donor and promotes solute carrier family 7 member 11 (SLC7A11)-mediated cystine uptake for GSH synthesis and subsequent inhibition of ferroptosis (203). In addition, NAPDH can also induce Trx and CoQ10-H biosynthesis to enhance the antioxidant barriers of cancer cells, thereby maintaining redox homeostasis in the TME (204–206). Tumour glycolysis is closely associated with the ferroptosis process. Various metabolites, such as lactate that is produced by tumour cells rely on the glycolytic pathway to reshape the homeostasis and metabolism of the microenvironment, resulting in higher lactate abundance. However, elevated lactate levels activate the hydroxycarboxylic acid receptor 1 (HCAR1) on the tumour cell membranes, which promotes the production of mono-unsaturated fatty acids (MUFA) and inhibits ferroptosis. Occurrence of the above process in the TME is achieved by lactate *via* the HCAR1/MCT1-sterol regulatory element-binding protein 1 (SREPB1)- stearoyl-coenzyme A (CoA) desaturase-1 (SCD1) signaling axis (207). The relationship between tumour cell ferroptosis and TME has yet to be conclusively determined. For instance, cancer cells with ferroptosis can induce immune cells in the TME to “eliminate” themselves by releasing damage-associated molecular patterns (DAMPs) signaling, acting as an immune-promoting factor (208, 209). At the same time, they can also promote cancer progression by releasing immunosuppressive signals, such as 8-OHdG and KRAS proteins that cause TAMs to differentiate into M2 phenotypes, as well as PGE2 that inhibits the functions of NK cells, DCs, and CD8(+) T cells (210–212). In determining how the TME affects tumour cells ferroptosis, it has been established that CD8(+) T cells, which produce IFN-γ by inhibiting the expressions of tumour cell system Xc- and reducing the synthesis of GSH, induces ferroptosis and inhibit tumour progression (213). The crosstalk between tumour cell ferroptosis and TME deserves further exploration. The link between PC glycolysis and ferroptosis and TME is shown in **Figure 3**.

## 6 Pancreatic cancer glycolysis and immune aging

**Figure 4:**
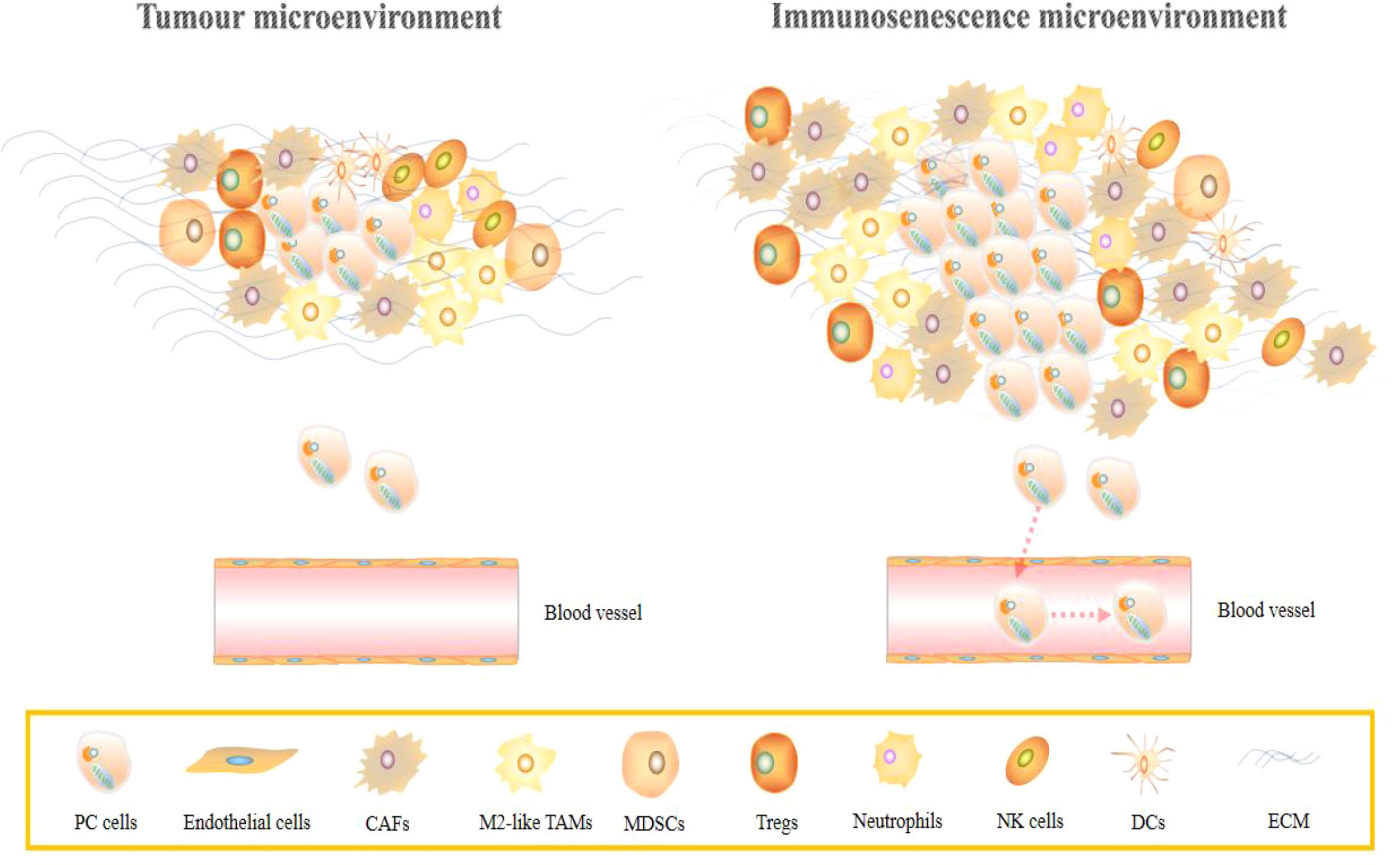
Schematic presentation of immune aging in PC. In a normal PC microenvironment, due to the presence of anti-tumour immune cells such as NK cells, the proliferation, invasion and vascular invasion of cancer cells are inhibited. However, in the immunosenescence microenvironment, the tumour suppressive abilities of immune cells are weakened, and rapid proliferation of cancer cells simultaneously induces massive infiltration of cancer-promoting immune cells, including Tregs as well as TAMs, and promotes cancer cell vascular invasion and distant metastasis.

A decrease in the ability of an organism to resist immune and a decrease in the functions of one or more organs usually increases in a time-dependent manner, a process referred to as senescence (214). Aging in organisms is attributed to increased cell senescence, and is characterized by cell cycle arrest and a persistent DNA damage response, which is an effective barrier against tumorigenesis but can also create an inflammatory environment that leads to cancer progression (215). Unlike immune senescence induced by the passage of time, tumour cells increase the glycolytic flux to resist the adverse effects of this senescence, and the large amounts of lactate produced promotes their own invasive and migratory abilities. This outcome results from increased activities of snail proteins, whose expression levels are up-regulated in a lactate dose-dependent manner and *via* the TGF-β1/SMAD axis. Lactate-induced snail protein also inhibits senescence by suppressing p16 and enhancing cell cycle progression (216). In a previous study, p16 was found to be inactive in 85% of PC models, confirming the reliability of the above conclusions (217). This is a change in cancer cells’ own metabolism to prevent aging, but whether similar changes occur in the TME to regulate cancer cell growth and development is unknown. IL-1α, which is over-expressed in the TME, induces KRAS mutations in PC patients, while IL-6 regulates STAT3 activation in PC cells and to drive cancer development (218, 219). However, these studies only focused on the changes in the epigenetic functions of cancer cells that were directly caused by senescent cells in the TME, such as proliferation and invasion, but rarely explored the underlying mechanisms, especially the connection with cancer cell metabolism (glycolysis) (220–223). Age, chronic inflammation, and changes in the microenvironment induce immune cell senescence. Senescent cells can remodel the microenvironment by secreting various cytokines, growth factors, chemokines, and senescence-associated secretory phenotypes (SASPs), ultimately leading to the development of malignant tumour development (224, 225). For instance, up-regulated Tregs cells in the TME induce DNA damage in CD4(+) T and CD8(+) T cells and overexpress senescence-associated β-galactosidase (SA-β-Gal), promoting cell cycle progression and growth arrest in the G0/G1 phase, leading to T cells senescence and impaired tumour immunosuppressive capacities, which are co-regulated by MAPK and STAT1/STAT3 signaling pathways (226). Liu et al. further found that among T cell subsets, Tregs have more active glucose and lipid metabolism rates. Their high glucose uptake capacities and accelerated depletion induces senescence and DNA damage in reactive T cells, which can be reversed by TLR signaling to enhance antitumour immunotherapy (226, 227). Secretion of IL-8 by Tregs that are enriched in malignant pleural effusions induces TAMs to produce TGF-β, which subsequently mediates c-Fos to regulate CCL22 secretion and further promotes Tregs recruitment, forming a vicious circle that promotes the development of an immunosuppressive microenvironment (228). Other immune cells, including NK cells, B lymphocytes, and DCs, all enter a state of senescence with age, resulting in tumorigenesis. However, studies have not established an association between inflammation, injury-induced immune aging and tumour metabolism. Recently, studies on “soil” and “seed” have become increasingly popular, especially in epithelial cancer cells. RAS oncogene activation and oxidative stress that are attributed to overproduction of ROS induce the upregulation of cancer-associated fibroblasts MCT4 (HIF-1α) in the TME, leading to decreased mitochondria function and increased rates of glycolysis, with consequent production of L-Lactate and ketone bodies that are taken up by cancer cells as an energy source for oxidative phosphorylation, thereby realizing the “metabolic symbiosis” between tumour cells and adjacent cells (229). In highly hypoxic TMEs, especially PC cells, can also take up biomacromolecules required for anabolism by adjacent cancer cells with high glycolytic flux (230). This provides support for cancer cells to use surrounding cells to rescue themselves from being susceptible to autophagy and senescence, and also opens up new directions for exploring the associations between immune senescence and tumour metabolism. A schematic presentation of PC immunosenescence is shown in **Figure 4**.

## 7 Metabolic therapy for pancreatic cancer

Since the “Warburg” effect was proposed, elucidation of malignant progression of tumours from a metabolic perspective will enhance the development of effective therapeutic options for cancer, particularly for cancers with extremely poor prognostic outcomes, such as PC. Metabolic therapy combined with TME allows PC patients to have better prognostic outcomes.

### 7.1 Targeted therapy

#### 7.1.1 Reduced glucose uptake and availability

Under chronic hypoxia and nutrient deficiency environments, efficient utilization of glucose is the main source of energy for tumour cells, and the increase in glucose uptake to enhance glycolytic enzymes activities is the key link in cancer proliferation and invasion. Therefore, the competitive utilization of glucose and the inhibition of glycolytic regulatory enzymes suppress the malignant proliferation of cancer cells. Activated T cells utilize aerobic glycolysis for their own growth and effector functions, but glucose deprivation or metabolic competition by tumour cells in the TME limits aerobic glycolysis of tumour-infiltrating T cells and inhibits T cells from producing effectors and antitumor responses. Immune function of CD4(+) T and CD8(+) T cells rely on the normal transduction of a large number of Ca+ and nuclear factors of activated T cells (NFAT) signals that require the maintenance of the glycolytic metabolite, phosphoenolpyruvate (PEP), which is difficult to achieve in an immunosuppressive microenvironment. Maintaining the stability of T cell glycolysis is crucial for inhibiting cancer cell proliferation. Phosphoenolpyruvate carboxykinase 1 (PCK1), as a key enzyme in gluconeogenesis, can increase the production of PEP in T cells, improve glucose uptake, and enhance its immune effects. Increasing the expressions level of PCK1 has become an effective target for tumour metabolic therapy (231). In the TME, T cells can activate PD-L1 on tumour cells by secreting IFN-γ. Cancer cells rely on PD1/PD-L1 interactions to reversely inhibit T cell functions to evade immune-mediated attacks. The above effects are achieved by PD-L1 promoting AKT/mTOR activities and glycolytic flux in cancer cells. In a previous study, after blocking PD-L1, the phosphorylation levels of AKT/mTOR target protein decreased, expressions of key glycolysis enzymes were down-regulated, the inhibitory effects of T cells were weakened, and the anti-tumour effects were finally enhanced (107). MDSC-mediated T cell suppression is closely associated with poor prognostic outcomes of tumour patients. In a hypoxic microenvironment, HIF-1α activates V-domain Ig suppressor of T cell activation (VISTA) in MDSCs, resulting in the above results. The synergistic effects of anti-VISTA and anti-PD-1 blockade improves the malignant processes in tumours. However, it has not been determined whether MDSCs directly lead to metabolic reprogramming in tumour cells (232). Apart from T cells, the possibility of targeting other components in the TME to affect glucose uptake and utilization by tumour cells should also be assessed.

#### 7.1.2 Inhibition of lactic acid content in TME

Low extracellular pH is usually a sign of solid tumours. Production of large amounts of lactic acid by glycolysis and glutamine metabolism in tumour cells results in its accumulation in the TME, which results in metabolic changes in fibroblasts, immune cells and endothelial cells in the TME, aggravation of TME acidity and promotion of immunosuppression. Lactate-induced excess secretion of IFN-γ activates c-Jun N-terminal kinase (JNK)/c-JUN, p38-MAPK signaling, and finally inhibits the normal functioning of cytotoxic T lymphocytes (CTL) (233). Tumour cells recruit monocytes and induce their differentiation into macrophages or DCs, but, the low pH environment in the TME inhibits the glucose uptake that is required for the DCs differentiation processes, turning it towards oxidative phosphorylation and promoting tumour progression. This process may be associated with the fact that the acidic environment promotes mTORC1 inhibition and ultimately reduces histone deacetylase (HDAC) activities (234). As the last enzymatic step in the glycolytic pathway, a key part of lactate production involves efficient functioning of lactate dehydrogenase, especially LDHA, which is crucial in tumour growth and metastasis, such as lung cancer, liver cancer, breast cancer and PCs (235–238). For tumours that are significantly LDH-dependent, as described above, genetic or pharmacological inhibition of LDHA effectively reduces cancer cell glycolytic flux and NADH/NAD ratio, and results in cancer cell apoptosis. Although the regulatory mechanisms of LDHA have been explored in some studies, he therapeutic significance of tumour lactate metabolism has yet to be established (237–239). In TME, the inhibition of LDHA also restricts cells with glycolysis as a preferential metabolic mode, such as Tregs, MDSCs and TAMs, which ultimately hinders the progression of tumours. However, there are some inconclusive findings, for instance, inhibition of LDHA suppresses IFN-γ expressions by T cells, which are worthy of further exploration (240). Export of lactate, which requires the regulation of lactate transporters (MCTs), is beneficial for preventing intracellular acidification and maintaining a high rate of glycolysis. In PC cells, knockdown of MCT1 and MCT4 expressions increased intracellular lactate accumulation and inhibited cell proliferation, migration as well as invasion (241). It has been postulated that CD147, gp70, and TMPRSS11B are associated with MCTs, but this has not been confirmed in PC models (242–244). Blockade of MCTs can also promote the activation of T cells and the secretion of IL-2 as well as IFN-γ (245). Diclofenac inhibits tumour growth and reduces the rate of lactate transport while suppressing the abundant of Tregs (246, 247). The above outcomes show that lactate metabolism is a potential therapeutic target in PC.

### 7.2 Immunotherapy

Hypoxia, low glucose, and low pH induces the directional differentiation of Tregs, MDSCs and M2 macrophages, and at the same time inhibites the functions of NK cells, CD4(+) T cells, CD8(+) T cells and DCs, and finally forms an immunosuppressive microenvironment, leading to tumour progression. Targeting tumour metabolism while inducing the functional recovery of immune cells in the TME is key in immunotherapy, especially for highly immunosuppressed pancreatic tumours. Jiang et al. assessed the association between glycolytic activities and immune signatures in 14 cancers and found that hyperglycolytic tumours exhibited better immunotherapeutic responses and favorable prognosis. This was attributed to increased expressions of PD-L1 by glycolysis, which confirms the importance of glycolytic activities in immunotherapeutic efficacy (105). In a breast cancer models, the abundant glycolytic activities of tumour cells promoted the expression of G-CSF and GM-CSF expressions, which was accompanied by T cell suppression and increased MDSC numbers. However, inhibition of the glycolytic process alleviates the tumour immunosuppressive state *via* AMPK-ULK1, autophagy pathway and CEBPB pathway (150). Targeted metabolic regulation of immunosuppression in PC cells is a topic for further research. As an important regulator of Wnt transduction in the canonical signaling pathway in tumours, Dickkopf-related protein 3 (DKK3) negatively regulates aerobic glycolysis [HK2, HIF-1α, GLUT-1, LDHA and 3-phosphoinositide-dependent protein kinase-1 (PDK-1)] in BxPC-3 cells. Moreover, glucose uptake and proliferation abilities of CD4(+) T cells co-cultured with BxPC-3 cells are also affected, but the molecular regulation mechanism has not been thoroughly explored (248). As an important branch of PC glycolysis, HBP not only promotes cancer cells proliferation and invasion, and also affects hyaluronic acid synthesis in the ECM and CD8(+) T cell infiltration degree. Reduction of hyaluronan and collagen production in the TME by inhibition of the rate-limiting enzyme glutamine-fructosamidyltransferase 1 (GFAT1) of HBP leads to extensive remodeling of the ECM and increased levels of CD8(+) T infiltration, while enhancing PD1 treatment responses, eventually resulting in tumour regression (249, 250). Interleukin 1 receptor-associated kinase 2 (IRAK2), which is highly expressed in PC, promotes the expressions of GLUT1, phosphoglucose isomerase (GPI1), and PDK1 by activating the immune-related pathway (NF-κB), thereby increasing glucose consumption and lactate production in cancer cells, providing bioenergetic and metabolic requirements for their survival and proliferation (251). However, there is a need to establish how it ultimately affects the immune status in the TME. Currently, immunotherapy for PC only shows the inhibition of immune checkpoints such as CTLA4, PD1 and PDL1, but the efficacy is average, thus, PC is also referred to as an immune cold tumour. There is a need to determine whether immunotherapeutic effects can be improved by targeting tumour metabolism.

### 7.3 Chemotherapy

Although postoperative chemotherapy is the preferred treatment option for PC patients, it has not been shown to effectively improve the prognosis of patients. Due to the increasing chemoresistance of PC, chemotherapy alone cannot promote the worsening prognosis of patients. Tumour glycolysis enhances chemotherapeutic drug resistance. Inhibition of key glycolysis enzymes, multidrug resistance gene-1 (MDR-1) and PKM-2 significantly increased the sensitivity of ovarian cancer cells to paclitaxel (252). The glycolysis inhibitor 2-Deoxy-D-glucose (2-DG) in PC increases glucose and lactate levels in the TME, but also increases cancer cell sensitivity to gemcitabine. Therefore, chemotherapy regimens combined with metabolism have attracted the attention of researchers (253). However, targeting tumour metabolism alone to optimize tumour chemotherapy is still not enough. Temporary changes in tumour metabolism cannot results in changes in the TME, and active ingredients in the TME still support tumour progression. Therefore, targeting tumour cell metabolism and changes in the TME to improve chemotherapeutic effects has become a priority. Upregulated purinergic receptor P2Y2 (P2RY2) expressions in PC can be activated by increasing the levels of TME-derived ATP. Activation of the PI3K/AKT/mTOR pathway by crosstalk with platelet-derived growth factor receptor-beta (PDGFRβ) leads to increased c-Myc and HIF-1α levels, leading to enhanced glycolysis. Blocking the efficient binding of extracellular ATP to P2RY2 and concomitant use of gemcitabine prolonged the survival time of xenografted PC mice (254). Apart from tumour cells, related CAFs, Tregs, TAMs, and MDSCs are also involved in TME acidification, while accumulation of acidic metabolites can also affect the functioning and viabilities of cancer cells (255). To overcome this challenge, cancer cells, including PC, overexpress enzymes that are involved in pH regulation, including carbonic anhydrase 9 (CA9) and CA12. Interestingly, incubation of PK-8 cells with gemcitabine alone significantly increased glycolysis and induced a decrease in mitochondrial functions, which may be one of the factors leading to PC cell drug resistance. Although the inhibition of CA9 did not affect the basal level of glycolysis, it significantly inhibited the gemcitabine-induced acceleration of extracellular acidification and mitochondrial functions in cancer cells, which was verified in xenografted mice. The combination of CA blocker and gemcitabine improved outcomes in PC mice models (256). Although these studies are few and weak, there is room for improvement. For instance, butyrate, a metabolite of beneficial microorganisms in the colon, inhibits glucose transport and glycolytic flux in colorectal cancer cells by reducing the abundance of enveloped GLUT1 and cytoplasmic G6PD *via* the GPR109A-AKT pathway. When combined use with 5-FU, it increases the efficacy of chemotherapy in cancer, implying that microorganisms can be targeted to enhance chemotherapeutic efficacies (257). Combination therapy with TME from the perspective of metabolism may improve the chemoresistance of PC cells. Studies should elucidate on the relationship between cancer cell metabolism, TME, and clinical drug efficacy.

### 7.4 Radiotherapy

The effects of radiotherapy on PC cells have yet to be conclusively determined, but there is no doubt that it provides an additional option for postoperative treatment of PC patients. Although less than 20% of patients with primary PC are sensitive to radiation, it is unable to effectively improve the patient’s very poor prognostic outcomes (258). High glycolytic activities in PC are inversely correlated with the effectiveness of radiation, and elevated DNA damage responses in cancer cells are involved in changes in the process (259–262). DNA damage repair in PC is inseparable from the mediation of MCU1, which also participates in the metabolic reprogramming of cancer cells, that is, mediates carbon generation of carbon in the PPP pathway to provide biological raw materials for nucleotide synthesis (263–265). In PC cell lines (HPAF2 and Capan2), overexpressed MUC1 reversed cancer cell sensitivity to radiation, which was caused by MUC1-mediated elevations in glucose uptake and glycolytic flux. The metabolites produced by the above process provide carbon raw materials for nucleotide biosynthesis, promote DNA damage repair, and inhibit cancer cells proliferation, but the changes in the TME are still unknown (266). Current research focuses on the effects of directly targeting tumour metabolism in radiotherapy. However, for PC, a tumour that is extremely insensitive to radiotherapy, treatment effect of this method have not been predicted. Signal transmissions in interactions between the TME and tumour metabolism are key targets to improve the efficacies of radiotherapy.

## 8 Summary and prospects

Despite advances in medical technology, a cure for PC has yet to be developed. Moreover, there are challenges that are associated with early diagnosis, high invasiveness, and chemotherapeutic resistance. However, the rise of proteomics and metabolomics offers hope for a cure of PC, especially glycolysis. As the main energy acquisition pathway for cancer cells in an extremely hypoxic environment, the inhibition of glycolysis will surely prevent cancer progression. For a highly malignant tumour like PC, targeting metabolism alone does not bring about a significant improvement in patient prognosis. Due to the metabolic heterogeneity and high plasticity of a single tumour itself, as well as the concomitant rearrangement of the TME, the crosstalk between the two induces the malignant progression of tumour cells. Interfering with the glycolytic pathway in PC from the perspective of TME may solve challenges associated with postoperative chemotherapeutic resistance, immune tolerance and ineffectiveness of radiotherapy.

Currently, research on the effect of the TME on tumour glycolysis is at its infancy, and several problems need to be solved, such as how metabolite based nutrient enrichment mechanisms cause metabolic reprogramming of cancer cells, such as epigenetic modification of histone lactic acidification due to lactic acid aggregation (195), how tumour metabolism affects the proportional distribution and metabolic differences of immunosuppressive cells in the TME, and how changes in microbiota composition affect tumour glycolysis to improve the efficacy of immunotherapy in patients. In addition, analysis of metabolic crosstalk with TME from a perspective of PC subtypes will facilitate the development of targeted interventions. The current clinical trial drugs only involve pure immune checkpoints, while related studies on tumour metabolism have only been effected in mouse models. These studies do not represent differences in tumour types caused by individual differences between patients. Moreover, due to the complexity of TME, the individualized regimen of combination therapy is more advantageous. In conclusion, targeting the combination of tumour metabolism and TME has great promise in improving the prognosis of PC patients.

## Author contributions

S.D.; Writing-Original draft preparation, Supervision, Project administration, W.L.; Writing- Original draft preparation, Visualization. X.L., Z.Z., Z.C., H.S., R.H. and C.C.; Writing - Review & Editing, Supervision. W.Z.; Writing-Original draft preparation, Supervision, Project administration, Funding acquisition. All authors have read and agreed to the published version of the manuscript.

## Glossary

**Table:**

PC	pancreatic cancer
TME	tumour microenvironment
GLUTs	glucose transporters
SGLTs	sodium-dependent active transporters
SWEETs	sugar transporters
PDAC	pancreatic ductal adenocarcinoma
OS	overall survival
PFS	progression-free survival
GEO	Gene Expression Omnibus data base
HK	Hexokinase
VEGF-A	Vascular endothelial growth factor A
PFKFB	phosphofructokinase
HIF	hypoxia-inducible factor
PPP	pentose phosphate pathway
TKT	transketolase
RPI	ribose 5-phosphate isomerase
RPE	ribulose 5-phosphate epimerase
F6P	fructose 6-phosphate
G3P	glyceraldehyde 3-phosphate
SHMT	serine hydroxymethyl transferases
THF	tetrahydrofolic acid
LKB1	lacking live kinase B1
PHGDH	phosphoglycerate dehydrogenase
O-GlcNAc	O-linked N-acetylglucosamine
HBP	hexosamine biosynthesis
GFPT1	glucose fructose-6-phosphate amidotransferase 1
OGT	O-GlcNAc transferase
OGA	O-GlcNAcase
CAFs	cancer-associated fibroblasts
TCA	tricarboxylic acid
MCT1	monocarboxylate transporter 1
LDHB	lactate dehydrogenase B
JAK2	Janus kinase 2
STAT3	signal transducer and activator of transcription 3
VEGFR	vascular endothelial growth factor receptor
Treg	regulatory T
TADC	tumour-associated DCs
IDO	indoleamine 2, 3-dioxygenase
GPX4	glutathione peroxidase 4
TAM	tumour-associated macrophages
CCL18	C-C motif chemokine ligand 18
VCAM-1	vascular cellular adhesion molecule-1
NF-κB	noncanonical nuclear factor-κB
EMT	epithelial-mesenchymal transition
TGF-β	transforming growth factor beta
NK	natural killer
IFN-γ	interferon-gamma
PK	pyruvate kinase
FBP1	fructose 1, 6-bisphosphatase 1
DCs	dendritic cells
MUC1	mucin 1
LPS	lipopolysaccharide
TLR3	toll-like receptor 3
ER	endoplasmic reticulum
MDSCs	myeloid-derived suppressor cells
AMPK	AMP-activated protein kinase
CEBPB	CCAAT/enhancer-binding protein beta
G-CSF	granulocyte colony-stimulating factor
GM-CSF	granulocyte macrophage colony-stimulating factor
PI3K	phosphatidylinositol 3-kinase
mTOR	mammalian target of rapamycin
CXCL1	C-X-C motif ligand 1
ERK1/2	extracellular signal-regulated kinase 1/2
Bregs	regulatory B cells
TSP1	thrombospondin 1
BAFF	B cell activating factor
BCR	B cell receptor
GLP1	glucagon-like peptide 1
G6PD	glucose-6-phosphate dehydrogenase
ROS	reactive oxygen species
GSSG	glutathione disulfide
GSH	glutathione
SLC7A11	solute carrier family 7 member 11
HCAR1	hydroxycarboxylic acid receptor 1
MUFA	mono-unsaturated fatty acids
RSL3	Ras-selective lethal small molecule 3
SREPB1	sterol regulatory element-binding protein 1
SCD1	stearoyl-coenzyme A (CoA) desaturase-1
DAMPs	damage-associated molecular patterns
SASPs	senescence-associated secretory phenotypes
SA-β-Gal	senescence-associated β-galactosidase
NFAT	nuclear factor of activated T cells
PEP	phosphoenolpyruvate
PCK1	phosphoenolpyruvate carboxykinase 1
VISTA	V-domain Ig suppressor of T cell activation
JNK	c-Jun N-terminal kinase
CTL	cytotoxic T lymphocytes
HDAC	histone deacetylase
MCT	lactate transporter
DKK3	Dickkopf-related protein 3
PDK-1	3-phosphoinositide-dependent protein kinase-1
GFAT1	glutamine-fructosamidyltransferase 1
IRAK2	interleukin 1 receptor-associated kinase 2
GPI1	phosphoglucose isomerase
MDR-1	multidrug resistance gene-1
2-DG	2-Deoxy-D-glucose
P2RY2	purinergic receptor P2Y2
PDGFRβ	platelet-derived growth factor receptor-beta
CA9	carbonic anhydrase 9
